# Positive psychotic symptoms as a marker of clinical severity in a transdiagnostic sample of help-seeking adolescents

**DOI:** 10.1007/s00787-024-02417-7

**Published:** 2024-03-30

**Authors:** Janko M. Kaeser, Stefan Lerch, Silvano Sele, Corinna Reichl, Julian Koenig, Ines Mürner-Lavanchy, Thomas Berger, Michael Kaess, Marialuisa Cavelti

**Affiliations:** 1 University Hospital of Child and Adolescent Psychiatry and Psychotherapy, University of Bern, Bolligenstrasse, 111, 3000 Bern, Switzerland; 2 Department of Child and Adolescent Psychiatry, Psychosomatics and Psychotherapy, Faculty of Medicine, University Hospital Cologne, University of Cologne, Cologne, Germany; 3 Department of Clinical Psychology and Psychotherapy, University of Bern, Bern, Switzerland; 4 Department of Child and Adolescent Psychiatry, Centre for Psychosocial Medicine, University of Heidelberg, Heidelberg, Germany

**Keywords:** Psychosis, Dimensional, Transdiagnostic, Adolescence, Youth

## Abstract

The present study aimed to examine the association between the presence, number, and type of positive psychotic symptoms (PPS) and clinical severity in adolescent patients. Five hundred-six patients aged 11–17 years were assigned to either the noPPS (n = 341), the delusional beliefs only (del; n = 32), the hallucinations only (hall; n = 80), or the delusional beliefs and hallucinations (del&hall; n = 53) group. Generalized Structural Equation Modeling was applied to identify the best-fitting model representing clinical severity indicated by psychiatric diagnoses, depressivity, personality pathology, non-suicidal self-injury, suicide attempts, perceived stress, and psychosocial impairments, assessed by interviews and questionnaires. The groups were compared concerning the final model’s factors. The final model consisted of three factors representing psychopathology and functional impairments, self-harming behavior, and perceived stress (BIC difference to reference model: 103.99). Participants with any PPS scored higher on all factors than the noPPS group (differences in SD: 0.49–1.48). Additionally, the del&hall group scored 1.31 SD higher on psychopathology and functional impairments than the hall group, and 1.16 SD higher on self-harming behavior compared to the del group. Finally, the hall group scored 0.84 SD higher on self-harming behavior than the del group, with no group differences in the other factors. In adolescent patients, the presence of PPS may represent a marker for a more severe form of mental disorder, with hallucinations being indicative of self-harming behavior. Early transdiagnostic assessment of PPS seems indicated as it may inform treatment in the context of clinical staging.

**Supplementary Information:**

The online version contains supplementary material available at 10.1007/s00787-024-02417-7.

## Introduction

Psychotic symptoms exist as a continuum in terms of severity, associated distress, and their effects on functioning and help-seeking behavior [1–4]. While psychotic experiences (PEs) occur across the lifespan, they are particularly prevalent in young people, with a median prevalence of 7.5% [5–9]. Adolescents with PEs are nearly three times more likely to be diagnosed with a non-psychotic disorder and four times more likely to be diagnosed with a psychotic disorder [10–12]. In addition, PEs in adolescence have been found to be associated with a greater number of co-occurring psychiatric diagnoses [6, 10], higher levels of depressive symptoms [13, 14], greater personality pathology [15], higher levels of subjectively perceived stress [16], and poorer social and global functioning [17–19]. A strong link with PEs has also been found with self-harming behavior [20–24], with individuals with PEs having shown threefold increased odds for non-suicidal self-injury (NSSI) and suicide attempts [25, 26], and fourfold increased odds of suicide death [27]. When the relationship between different PEs subtypes (e.g., visual and auditory hallucinations, mind reading, referential ideas, and persecutory delusions) and self-harming behavior was examined, only auditory and visual hallucinatory experiences remained significant predictors after controlling for confounding factors, such as depression [21, 28]. Overall, PEs during adolescence are seen as a non-pathognomic risk maker for negative long-term outcomes, including mental health problems and functional impairments [12, 29, 30]. Accordingly, identifying those young people with such markers or early signs of mental disorder is a crucial precondition for indicated prevention and early intervention efforts in order to prevent negative long-term outcomes. A key shortcoming of the current empirical evidence on the clinical significance of PEs in adolescents is that it is mainly community-based, relying on potentially inflated self-reports [31]. Consequently, it is still unclear whether psychotic symptoms are also an indicator of greater clinical severity in help-seeking youth, where they could inform treatment selection and planning in the context of a clinical staging approach [32]. Given the rising demand and decreasing resources for youth mental health services, understanding the maximum intensity of treatment needed for each individual is crucial. To close this gap, the current study aimed at examining the link between the presence, the number of, and the type of positive psychotic symptoms (PPS; delusional beliefs and hallucinations) and clinical severity in a help-seeking, transdiagnostic sample of adolescents. We applied an explorative latent modeling approach that allowed us to consider multiple indicators of clinical severity (e.g., number of psychiatric diagnoses, psychosocial impairments, self-harming behavior, etc.) simultaneously in a single statistical model and to explore the impact of psychotic symptoms on the factor structure that represents clinical severity. Based on previous research, we hypothesized that (1) individuals with hallucinations and/or delusional beliefs would show greater clinical severity than individuals without PPS [10–19], (2) that individuals with both hallucinations and delusional beliefs would show greater clinical severity than individuals with only hallucinations or delusional beliefs [6], and (3) that individuals who experience hallucinations only would particularly show more self-harming behavior than individuals who experience delusional beliefs only [21, 28].

## Methods

### Participants and procedures

The sample of this study (N = 506) stems from two cohort studies conducted at the University Hospital of Child and Adolescent Psychiatry and Psychotherapy, University of Bern, Switzerland. Participants were consecutively recruited from general psychiatric inpatient and day-care services (“Bernese Basic Documentation” [BeBaDoc] sample), and a specialized outpatient clinic for risk-taking behavior and self-harm (“Ambulanz für Risikoverhalten und Selbstschädigung” [AtR!Sk]) sample). Inclusion criteria were: 11–18 years of age (BeBaDoc sample), 12–17 years of age (AtR!Sk sample), self-harming or risk-taking behavior (AtR!Sk sample), and sufficient fluency in German language skills. Exclusion criteria were patients lacking capacity to understand the study details or provide informed consent. Written informed consent was obtained from all participants, as well as from a parent or legal guardian for those under the age of 14 years. Assessments were conducted by trained clinicians (AtR!Sk) or trained doctoral and psychology students (BeBaDoc). In the AtR!Sk study, assessments formed part of the routine diagnostic assessment procedure at entry to the clinic and therefore participants were not reimbursed. BeBaDoc participants received the equivalent of 20CHF worth of vouchers. Both studies were approved by the local ethics committee (BeBaDoc Ethics ID: 2018–01339; and AtR!Sk Ethics ID: 2018–00942).

### Measures

#### Demographic data

Information was collected using a standardized set of interview questions to assess age, sex, and school type.

####  Assessment of psychiatric diagnoses and positive psychotic symptoms (PPS)

The Mini International Neuropsychiatric Interview for Children and Adolescents (MINI-KID; [33]) was used to rate current psychiatric disorders. Diagnosis was included in the analyses as a categorical variable with three levels: no diagnosis, one diagnosis, and two or more diagnoses. In addition, specific items from the section on assessment of psychotic disorders and affective disorders with psychotic features were used to assess delusional beliefs (present at the time of the interview) and hallucinations (occurrence in the past month; a full list of items is provided in the Supplementary Material [SM]). A group variable for the occurrence of PPS with the following mutually exclusive categories was created: no occurrence of PPS (noPPS), delusional beliefs only (del), hallucinations only (hall), and co-occurring delusional beliefs and hallucinations (del&hall). Borderline personality disorder (BPD) was assessed using the corresponding section of the Structured Clinical Interview for DSM-IV Axis II (SCID-II) [34, 35].

#### Personality functioning

The Semi-structured Interview for DSM-5 Personality Functioning (STiP-5.1; [36]) assesses impairment in self- and interpersonal functioning according to the alternative DSM-5 model for personality disorders (AMPD; [37]). The interviewer scores the degree of impairment in each facet on the Personality Functioning Scale (LPFS; [38]), ranging from 0 (no impairment) to 4 (extreme impairment). For the analyses, the mean score of the two domain scores (self- and interpersonal functioning) was used, which in turn were composed of the mean of their underlying three facets each.

#### Depression severity

The Children’s Depression Rating Scale (CDRS; [39]), a 16-item semi-structured interview, was used to determine the severity of depression. Items were rated on a scale of 1 (no particular problems) to 7 (severe, clinically significant problems), respectively 1 to 5 for the items on sleep, appetite, and speech rate. The sum score was used for analyses.

#### Psychosocial functioning

The Social and Occupational Functioning Assessment Scale (SOFAS; [40]) was used in the AtR!Sk sample and the Children’s Global Assessment Scale (CGAS; [41]) in the BeBaDoc sample. Both are clinician-rated measures of global functional capacity, with scores ranging from 0 (minimum functional capacity) to 100 (maximum functional capacity). For the analyses, scores were standardized.

#### Perceived stress

The Perceived Stress Scale (PSS; [42]) was used to assess non-specific stress. Items were rated on a score from 0 (never) to 4 (very often). The sum score ranging range from 0 to 40 was used for analyses.

#### NSSI and suicide attempts

According to the DSM-5, NSSI is the intentional damage of body tissue without suicidal intention for socially unsanctioned purposes; and a suicide attempt is a self-initiated sequence of behaviors by an individual who, at the time of initiation, expects that the set of actions would lead to his or her own death [37]. The Self-Injurious Thoughts and Behaviors Interview (SITBI) was used to assess the number of days with NSSI incidents and the frequency of suicide attempts in the last year [43]. NSSI incidents in the last year were included in the analyses as a categorical variable with three levels according to criterion A of the DSM-5 diagnosis of Non-suicidal Self-Injury (i.e., no incidents, NSSI on one to 4 days [subthreshold], NSSI on 5 or more days [criterion A fulfilled]; [37]). Suicide attempts in the last year were operationalized with a three-level categorical variable (i.e., no attempts, one attempt, two or more attempts).

### Statistical analyses

Missing value analyses revealed less than 5% missing values in all variables. We performed available-case analyses. To examine clinical severity at the latent level, we first fitted a structural equation model (SEM) with one single factor overarching all indicators (i.e., depressivity, psychosocial functioning, perceived stress, personality functioning, suicide attempts, NSSI incidents, number of diagnoses) as a baseline model. Its global model fit was evaluated using the Comparative Fit Index (CFI), the Standardized Root Mean Square Residuals (SRMR), and the Root Mean Square Error of Approximation (RMSEA) [44]. The SEM was then compared to a Generalized Structural Equation Model (GSEM) with the same model structure, as it is better suited for ordinal data present in three of the indicators (i.e., number of diagnoses, NSSI incidents, and suicide attempts; [45]). Finally, a GSEM, including the PPS group variable and covariates (i.e., sex, age, sample [AtR!Sk, BeBaDok]) loading as predictors on the factor, was fitted and compared with the simple GSEM, using the Likelihood-Ratio Test. To determine if a more complex factor structure could more accurately explain the data, we tested every possible 2- and 3-factor GSEM model structure with the seven designated indicators (n = 560). Analyses were performed with 1000 iterations for each model structure with covariates and the dummy coded PPS group variable loading on every latent factor. Given seven indicators, we limited our analysis to models with no more than three factors to maintain analytical parsimony. The error terms of the factors were allowed to correlate because a common measurement error was expected. The best-fitting model of all possible factor combinations, selected for its lowest Bayesian Information Criterion (BIC), provided the factor composition that was contrasted between the groups to answer our research questions. To test the hypotheses, differences in the latent factor(s) of the best-fitting model between the PPS groups were examined using contrasts (hypothesis 1 [presence]: noPSS versus PPS [i.e., del, hall, del&hall]; hypothesis 2 [number]: del&hall vs. del, del&hall vs. hall; hypothesis 3 [type]: del vs. hall). Group differences in standard deviation (DSD) with confidence intervals were calculated as effect size measures [46], with a DSD of at least 0.5 being considered as clinically relevant [47]. To assess if psychotic disorders influenced the effects, we recalculated the best-fitting model and contrasts after excluding patients with a psychotic disorder (ICD-10 F2 diagnosis; sensitivity analysis). Analyses were performed in Stata 17 [48] and Mplus (Version 8.10; [49]). An alpha level of 0.05 was applied.

## Results

### Sample characteristics

Of 643 patients who were eligible (BeBaDoc: n = 344, 53, 5%; AtR!Sk: n = 299, 46.5%), 75 (11.7%) did either not give informed consent or dropped out after consent (BeBaDoc: n = 42, 6.5%; AtR!Sk: n = 33, 5.1%). In addition, 42 (6.5%) participants were excluded because of duplicate measurements or participation in both studies (BeBaDoc: n = 22, 3.4%; AtR!Sk: n = 20, 3.1%). Of the 526 remaining participants, 20 (3.1%) were excluded due to missing data on the PPS group variable, leaving a total of N = 506 (78.7%) for analyses. Sociodemographic and clinical characteristics are reported in Table 1.

**Table 1:** Sociodemographic and Clinical Characteristics

	Total	Presence of PPS			
		noPPS (*n* = 341)	del (*n* = 32)	hall (*n* = 80)	del&hall (*n* = 53)
	*M* (*SD*)/*n* (%)	*M* (*SD*)/*n* (%)	*M* (*SD*)/*n* (%)	*M* (*SD*)/*n* (%)	*M* (*SD*)/*n* (%)
Age (years)	15.42 (1.52)	15.35 (1.58)	15.47 (1.41)	15.54 (1.32)	15.62 (1.51)
Sex					
Female	400 (79.05)	266 (78.01)	28 (87.50)	65 (81.25)	41 (77.36)
Male	106 (20.95)	75 (21.99)	4 (12.50)	15 (18.75)	12 (22.64)
Highest level of education^a^					
Primary school (ISCED levels 0–1; at least 6 school years)	72 (14.26)	47 (13.82)	8 (25.00)	12 (15.00)	5 (9.43)
Secondary school (ISCED level 2; 9–10 school years)	351 (69.50)	230 (67.65)	19 (59.38)	60 (75.00)	42 (79.25)
High school (ISCED level 3; 12–13 school years)	79 (15.64)	60 (17.65)	5 (15.63)	8 (10.00)	6 (11.32)
Other	3 (0.59)	3 (0.88)	0 (0.00)	0 (0.00)	0 (0.00)
Intake of neuroleptics (current)					
No	433 (85.74)	301 (88.53)	27 (84.38)	66 (82.50)	39 (73.58)
Yes	72 (14.26)	39 (11.47)	5 (15.63)	14 (17.50)	14 (26.42)
Dataset					
AtR!Sk	244 (48.22)	168 (49.27)	10 (31.25)	43 (53.75)	23 (43.40)
BeBaDoc	262 (51.78)	173 (50.73)	22 (68.75)	37 (46.25)	30 (56.60)
Depression Severity	52 (16.39)	49 (15.57)	61 (18.49)	54 (13.89)	63 (16.58)
Psychosocial functioning^b^	0.00 (1.00)	0.12 (1.00)	−0.30 (0.95)	−0.04 (0.93)	−0.58 (0.90)
Stress	26.03 (6.62)	24.87 (6.81)	28.55 (5.64)	28.11 (5.60)	28.88 (5.30)
Personality functioning	1.17 (0.72)	1.05 (0.68)	1.56 (0.93)	1.27 (0.71)	1.54 (0.67)
Suicide attempts (last year)					
No attempts	341 (68.20)	252 (74.12)	22 (73.33)	43 (54.43)	24 (47.06)
One attempt	57 (11.40)	35 (10.29)	2 (6.67)	15 (18.99)	5 (9.80)
Two or more attempts	102 (20.40)	53 (15.59)	6 (20.00)	21 (26.58)	22 (43.14)
NSSI criterion ‘A’ DSM-5					
No NSSI incidents	104 (20.84)	82 (24.19)	8 (26.67)	7 (8.97)	7 (13.46)
Subthreshold (1–4 incidents)	46 (9.22)	31 (9.14)	7 (23.33)	6 (7.69)	2 (3.85)
Criterion fulfilled	349 (69.94)	226 (66.67)	15 (50.00)	65 (83.33)	43 (82.69)
Number of diagnoses					
No diagnoses	73 (14.43)	62 (18.18)	2 (6.25)	7 (8.75)	2 (3.77)
One diagnosis	104 (20.55)	88 (25.81)	2 (6.25)	10 (12.50)	4 (7.55)
Two or more diagnoses	329 (65.02)	191 (56.01)	28 (87.50)	63 (78.75)	47 (88.68)
Mental, Behavioral and Neurodevelopmental disorders					
F10-F19	73 (15.70)	43 (13.78)	4 (13.33)	16 (22.22)	10 (19.61)
F20-F29	22 (4.73)	1 (0.32)	4 (13.33)	10 (13.89)	7 (13.73)
F30-F39	384 (82.58)	250 (80.13)	27 (90.00)	61 (84.72)	46 (90.20)
F40-F48	297 (63.87)	188 (60.26)	24 (80.00)	49 (68.06)	36 (70.59)
F50-F59	61 (13.12)	43 (13.78)	6 (20.00)	5 (6.94)	7 (13.73)
F60-F69	54 (11.61)	33 (10.58)	3 (10.00)	7 (9.72)	11 (21.57)
F80-F89	33 (7.10)	25 (8.01)	3 (10.00)	1 (1.39)	4 (7.84)
F90-F98	87 (18.71)	51 (16.35)	5 (16.67)	19 (26.39)	12 (23.53)

N = 506. noPPS = no occurrence of positive psychotic symptoms, del = delusional beliefs only, hall = hallucinations only, del&hall = delusional beliefs and hallucinations. M = mean, SD = standard deviation

^a^Education levels are based on ISCED

^b^Standardized values

^c^No F0 or F7 disorders were diagnosed

### Latent representation of clinical severity

The 1-factor SEM performed acceptable (RMSEA = 0.074; 90% confidence interval [CI]: 0.053, 0.096) to excellent (CFI = 0.961; SRMR = 0.041; [50, 51]). The GSEM, better suited for ordinal data [45], showed similar results to the SEM (see Table 2). Finally, the likelihood ratio test comparing the 1-factor GSEM with and without predictors was significant, indicating that the inclusion of the predictors significantly improved the model fit (χ^2^ [6] = 116.20, p < 0.001).

**Table 2:** Comparison between SEM and GSEM (without predictors)

	1-factor models			
	SEM		GSEM	
	Coeff	SE	Coeff	*SE*
Depression severity^a^	0.83***	0.04	0.83***	0.04
Psychosocial functioning^a^	− 0.67***	0.04	− 0.67***	0.04
Stress^a^	0.61***	0.04	0.61***	0.04
Personality functioning^a^	0.70***	0.04	0.71***	0.04
Suicide attempts^b,c^	0.38***	0.04	1.29***	0.16
NSSI incidents^b,c^	0.32***	0.04	0.93***	0.14
No. of diagnoses^b,c^	0.44***	0.03	1.79***	0.19
CFI	961	−	−	−
SRMR	041	−	−	−
RMSEA^d^	074	−	−	−
AIC	8134.07	−	7398.69	−
BIC	8222.11	−	7488.26	−
χ^2^	50.98***	−	−	−

N = 489. Since SEM deletes listwise in case of missing data, complete-case analysis was performed for both models to allow comparison. CFI, SRMR, RMSEA and χ^2^ are not available for the GSEM

^a^Standardized effect of continuous variable

^b^Non-standardized effect of ordinal variable

^c^Within GSEM: Coefficient in log odds calculated using ordered logistic regression

^d^90%-confidence interval [0.053, 0.096]

^***^ indicates *p* < 001

**Fig. 1:**
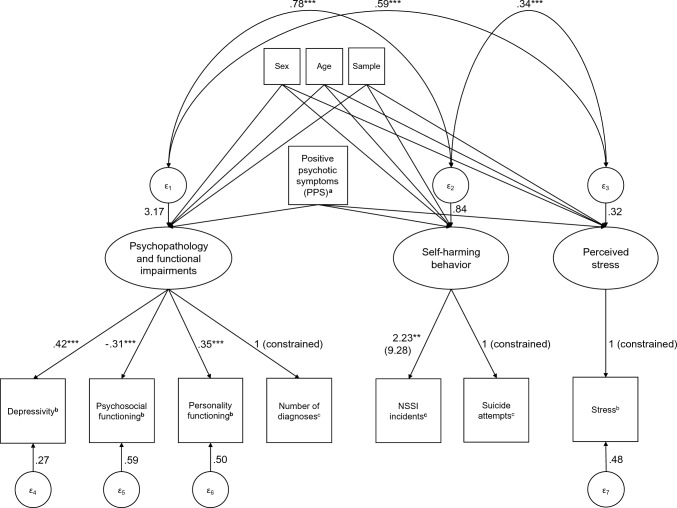
The best-fitting multifactorial model (GSEM) with three factors. N = 506. Coefficients are unstandardized unless otherwise stated. Path coefficients of the control variables Sex, Age, and Sample have been omitted for presentation reasons. Covariances of the latent factors are standardized. Complete results can be found in SM. ^a^Coefficients of the three dummy-coded variables representing the PPS group variable have been omitted from this figure for simplicity, as they do not allow any meaningful interpretation. ^b^Standardized coefficients. ^c^Coefficient in unstandardized log odds calculated using ordered logistic regression. Odds ratio in parentheses. *** indicates* p* < .001*,* ** indicates p < .01

The exploratory analyses of all possible 2- and 3-factor models given the seven selected indicators showed that using the BIC as criterion for good model fit, a 3-factor model appeared to fit the data best (BIC: 7183.84). This best-fitting 3-factor model showed a difference of more than ten relative to the 1-factor GSEM (BIC: 7287.83) and the next best convergent multifactorial model (BIC: 7222.34), indicating very strong evidence for better model fit [52, 53]. The factor structure we derived through this model selection process can be seen in Fig. 1, where we labeled the three factors based on their indicators as psychopathology and functional impairments, self-harming behavior, and perceived stress. The factors’ error terms were moderately to strongly correlated with each other. See SM for the complete results for the 1-factor and the best-fitting 3-factor GSEM. As Stata’s GSEM module did not allow the necessary parameter restriction of the Stress factor to estimate its error variance in an interpretable way (setting error variance of manifest indicator to zero or including manifest indicator instead of factor not possible), we fitted an equivalent model in Mplus, where Stress was included as a manifest variable. This model showed the same loglikelihood (H0 value =  − 3461.17; see SM for complete results), confirming the validity of the final model.

### Hypotheses testing

When testing hypothesis 1, adolescents with PPS (del, hall, or del and hall) scored significantly higher on psychopathology and functional impairments (DSD = −1.48, 95% CI − 1.99, −0.98), self-harming behavior (DSD =  − 0.49, 95% CI − 0.84, − 0.14), and perceived stress (DSD =  − 0.53, 95% CI  − 0.70, − 0.35) compared with adolescents without PPS (see Table 3 for contrasts and SM for a graphical representation of their magnitude). Additional exploratory post-hoc analyses revealed that adolescents with hallucinations only had significantly higher scores on all three factors (psychopathology and functional impairments: DSD =  − 0.79, 95% CI − 1.31, − 0.27; self-harming behavior: DSD =  − 0.66, 95% CI − 1.10, − 0.23; perceived stress: DSD =  − 0.44, 95% CI − 0.66, − 0.22), while those with delusional beliefs only showed higher scores on psychopathology and functional impairments (DSD = -1.56, 95% CI −2.38, − 0.75) and perceived distress (DSD =  − 0.54, 95% CI − 0.87, -0.21) but not self-harming behavior when compared with those without PPS.

**Table 3:** Group comparisons of the factor values of the best-fitting multifactorial model

	Psychopathology and functional impairments		Self-harming behavior		Perceived stress	
	Difference in *SD*	95% CI	Difference in *SD*	95% CI	Difference in *SD*	95% CI
H1						
noPPS vs. PPS	− 1.48***	− 1.99, − 0.98	− 0.49**	− 0.84, − 0.14	− 0.53***	− 0.70, − 0.35
noPPS vs. del	− 1.56***	− 2.38, − 0.75	0.17	− 0.35, 0.70	− 0.54**	− 0.87, − 0.21
noPPS vs. hall	− 0.79**	− 1.31, − 0.27	− 0.66**	− 1.10, − 0.23	− 0.44***	− 0.66, − 0.22
H2						
del vs. del&hall	− 0.53	− 1.43, 0.37	− 1.16**	− 1.95, − 0.36	− 0.06	− 0.47, 0.34
hall vs. del&hall	− 1.31**	− 2.06, − 0.56	− 0.32	− 0.91, 0.27	− 0.17	− 0.48, 0.15
H3						
del vs. hall	0.77	− 0.07, 1.62	− 0.84*	− 1.50, − 0.18	0.10	− 0.27, 0.48

N = 506. SD = Standard deviation from factor value estimated for the linear combinations of the dummy-coded PPS group variable, (no) PPS = (no) occurrence of positive psychotic symptoms, del = delusional beliefs only, hall = hallucinations only, del&hall = delusional beliefs and hallucinations, CI = confidence interval

***indicates p < .001, ** indicates p < .01, * indicates p < .05

Testing hypothesis 2 revealed that adolescents with both delusional beliefs and hallucinations demonstrated comparable scores of psychopathology and functional impairments and perceived stress, but greater scores of self-harming behavior (DSD =  − 1.16, 95% CI  − 1.95, − 0.36), compared with adolescents with delusional beliefs only. In contrast, when compared to adolescents with hallucinations only, adolescents with both delusional beliefs and hallucinations scored higher on psychopathology and functional impairments (DSD =  − 1.31, 95% CI − 2.06, − 0.56), with comparable scores for self-harming behavior and perceived stress. Finally, the examination of hypothesis 3 showed that adolescents with hallucinations only had a higher self-harming behavior score than adolescents reporting delusional beliefs only (DSD =  − 0.84, 95% CI − 1.50, 0.18). In contrast, no group differences were found for psychopathology and functional impairments, and perceived stress. To better understand the group difference in self-harming behavior, we additionally explored whether participants with delusional beliefs only and those with hallucinations only differed in NSSI and suicide attempts, respectively, using two separate ordered logistic regressions. Adolescents experiencing hallucinations only were 4.29 (95% CI 1.61, 11.45) times more likely to report more frequent NSSI than individuals with delusional beliefs only (Model: Chi2 [6] = 148.03, p < 0.001, n = 499; see SM for complete results). No significant group difference was found for suicide attempts (OR = 2.12, p = 0.108; Model: Chi2 [6] = 31.86, p < 0.001, n = 500; see SM for complete results). The sensitivity analysis revealed no differences in results when participants with a psychotic disorder were excluded.

## Discussion

This study examined PPS as a marker of clinical severity in a help-seeking, transdiagnostic sample of adolescents using a latent modeling approach. Four main findings emerged: First, clinical severity was best represented by three latent factors; psychopathology and functional impairments overarching general indices of psychopathology (i.e., depressivity, personality functioning, and number of psychiatric diagnoses) and impairments in psychosocial functioning, self-harming behavior represented by NSSI and suicide attempts, and perceived stress. The factor psychopathology and functional impairments is consistent with the p factor; a latent dimension that represents covariation among all forms of psychopathology [54]. Recent studies suggest that the p factor may also be relevant for the understanding of psychopathology in children and adolescents [55–57], and that personality pathology can be meaningfully included alongside general psychopathology in a joint personality-psychopathology framework in youth [58]. The differentiation of the self-harming behavior from the psychopathology and functional impairments factor is in line with evidence indicating that self-harm occurs across a variety of internalizing and externalizing psychopathology and personality pathology, and may thus represent a transdiagnostic marker of greater clinical severity [59]. Moreover, the two components of this factor, NSSI and suicide attempts, show shared etiological factors, biological correlates, and genetic overlap [60]. The identification of a perceived stress factor may reflect that the experience of general subjective distress is different from the other psychopathology- and risk-based indices of clinical severity. Another reason could be stress being the only construct measured through self-report [61]. Second, in comparison to adolescents without PPS, those with at least one PPS showed higher scores on all factors (except for the delusions only group, which did not differ in self-harming behavior compared to the no PPS group). Third, adolescents with two PPS scored higher on psychopathology and functional impairments when compared to those with hallucinations only, and higher on self-harming behavior when compared to those with delusional beliefs only. Fourth, adolescents with hallucinations or delusional beliefs only differed from each other only regarding self-harming behavior, with higher scores for the former group. Post-hoc analyses revealed that this group difference was mainly based on more frequent NSSI in the stand-alone hallucinations group, with no group difference in the frequency of suicide attempts. The effect sizes for the group differences were at or above the threshold of 0.5 SD, supporting the clinical relevance of the findings. Taken together, these findings extend previous community-based research that found PE during adolescence to be a risk maker for negative long-term outcomes (e.g., mental health problems and functional impairments) by demonstrating that in help-seeking adolescents the presence of PPS indicates a more severe form of mental disorder, with hallucinations being particularly indicative of an increased risk for self-harming behavior such as NSSI. The fact that the results remained stable when adolescents with psychotic disorders were removed from the analyses underscores the transdiagnostic relevance of the findings and is consistent with the hypothesis that psychotic thought processes are a sign of peak severity [54]. Thereby, adolescents experiencing both hallucinations and delusions appear to lie at the top end of the clinical severity continuum, which may not just be due to a coincidental cumulative effect of an additional psychotic symptom, but to a specific dynamic caused by higher etiological loading (i.e., higher levels of exposure to genetic and environmental risk factors). This interpretation is supported by studies showing that the co-occurrence of hallucinations and delusions is more common than expected by chance [62–64]. The differential results for hallucinations and delusional beliefs confirm earlier community-based findings demonstrating hallucinatory experiences being more consistently linked to self-harming behavior than other types of PPS [28, 65, 66]. Notably, auditory hallucinations in clinical samples have more negative content (e.g., commands to harm oneself) compared to non-clinical samples [67], substantially increasing the risk for self-harming behavior [65, 68, 69]. Strengths of this study include the large sample consisting of adolescent in- and outpatients of the primary public mental health service in the region; the comprehensive diagnostic assessment through standardized clinical interviews conducted by highly trained researchers or mental health professionals; the latent modeling approach allowing to simultaneously examine the relationship between several indicators of clinical severity and PPS that has to date mostly been examined in separate studies; and the consideration of personality pathology along with general psychopathology, self-harming behavior, perceived stress, and psychosocial functioning as indicators of clinical severity. In accordance with the new dimensional models for personality disorders outlined in DSM-5 and ICD-11 [37, 70], both the level of impairment in personality functioning (STiP-5.1) and the diagnostic criteria for BPD (SCID-II) were assessed. While the diagnosis of personality disorders in adolescents remains a subject of ongoing debate [71–73], compelling evidence suggests that (B) PD is reliable and valid diagnosis in adolescents and that early detection and intervention can exert a profound influence on the life trajectories of the affected young people, underscoring the significance of timely identification and support [74–76]. We also acknowledge several limitations: First, PPS were assessed using the MINI-KID, which is not a specialized instrument for psychotic symptoms or disorders and did not allow for the differentiation of variants of PPS. Second, due to the relatively small sample sizes of the group with only delusions (del; n = 32) and the group with delusional beliefs and hallucinations (del&hall; n = 53), smaller group differences may not have reached statistical significance due to low statistical power (Type II error). Third, although the BIC we used as criterion for model selection, which aims to strike a balance between goodness of fit and overfitting through a penalty term for the number of parameters [52], it does not eliminate the possibility of choosing an incorrect model [77]. We acknowledge that it can’t be ruled out that the high correlation of the factor’s error terms could be a sign of overfitting and want to emphasize that the latent modeling approach was explorative, meaning that the identified factor structure needs to be validated in other clinical samples to ensure robustness and generalizability. Finally, since we did not test for multiple hierarchies, we cannot rule out that a latent model with several hierarchical levels would better fit the data than the identified three-factor model. Future longitudinal studies are warranted to investigate the association between PPS and clinical severity over time, applying gold-standard measures of psychotic symptoms for clinical populations.

To conclude, we found PPS being a marker for greater clinical severity in help-seeking adolescents, with the presence of hallucinations being indicative for increased risk of self-harm, particularly NSSI. Clinically, this demonstrates the need for routine transdiagnostic assessment of psychotic symptoms, as it can help to identify youth with a more severe form of mental disorder and risk for self-harm who are in need of more intense treatment. Thus, in the context of clinical staging models, psychotic symptoms may serve as a transdiagnostic marker that informs treatment selection and planning, and improves allocation of limited resources in adolescent mental health services [32, 78].

## Supplementary Information

Below is the link to the electronic supplementary material.

Supplementary file1 (PDF 572 KB)

## Data Availability

Can be requested from the corresponding author.

## Code availability

Can be requested from the corresponding author.
