# Supplementary material for: Positive psychotic symptoms as a marker of clinical severity in a transdiagnostic sample of help-seeking adolescents
Source: Eur Child Adolesc Psychiatry. 2024 Mar 30;33(10):3637–47. doi: 10.1007/s00787-024-02417-7 (PMC11564335; doi:10.1007/s00787-024-02417-7)
Supplement: Supplementary file 1 — Supplementary file1 (PDF 572 KB) [file 787_2024_2417_MOESM1_ESM.pdf]

Supplementary Materials for:

Positive psychotic symptoms as a marker of clinical severity in a transdiagnostic sample of help-seeking adolescents

Submitted to:

European Child and Adolescent Psychiatry

Janko M. Kaeser[1], Stefan Lerch[1], Silvano Sele[1], Corinna Reichl[1], Julian Koenig[2], Ines Mürner-Lavanchy[1], Thomas Berger[3], Michael Kaess[1,4], Marialuisa Cavelti[1]

[1] University Hospital of Child and Adolescent Psychiatry and Psychotherapy, University of Bern, Bolligenstrasse 111, 3000 Bern 60, Switzerland

[2] University of Cologne, Faculty of Medicine and University Hospital Cologne, Department of Child and Adolescent Psychiatry, Psychosomatics and Psychotherapy, Cologne, Germany

[3] Department of Clinical Psychology and Psychotherapy, University of Bern, Bern, Switzerland

[4] Department of Child and Adolescent Psychiatry, Centre for Psychosocial Medicine, University of Heidelberg, Heidelberg, Germany

Corresponding author: Marialuisa Cavelti, University Hospital for Child and Adolescent Psychiatry and Psychotherapy, Bolligenstrasse 111, 3000 Bern 60, Switzerland, marialuisa.cavelti@unibe.ch, ORCID: 0000-0003-4726-396X

Content:

- Table 1: Results of the 1-factor GSEM including predictors
- Table 2: Results of best-fitting 3-factor GSEM
- Table 3: Results of the model computed in Mplus corresponding to the best-fitting 3-factor GSEM, with stress included as manifest variable in the factor structure
- Table 4: Results of Ordered Logistic Regression with *NSSI* as outcome (additional exploration of hypothesis 3)
- Table 5: Results of Ordered Logistic Regression with *suicide attempts* as outcome (additional exploration of hypothesis 3)
- Table 6: Dummy coding of the PPS group variable
- Group comparisons of the factor values of the best-fitting multifactorial model: An illustration of the magnitude of the contrasts
- Items of the Mini International Neuropsychiatric Interview for Children and Adolescents (MINI-KID; Sheehan et al., 2010) used to assess positive psychotic symptoms (translated from German to English)

Table 1
Results of the 1-factor GSEM including predictors

		Loadings of the factor on the indicators					
		Coeff.	*SE*	z	P	95% CI	
Depression severity[a]	Factor loading	0.741	0.040	21.32	0.000	0.673	0.809
	Constant	-0.754	0.384	-1.96	0.050	-1.507	0.000
Psychosocial functioning[a]	Factor loading	-0.569	0.038	-14.99	0.000	0.644	-0.495
	Constant	0.577	0.298	1.94	0.053	0.007	1.161
Stress[a]	Factor loading	0.536	0.038	14.06	0.000	0.461	0.611
	Constant	-0.545	0.281	-1.94	0.053	-1.097	0.007
Personality functioning[a]	Factor loading	0.611	0.037	16.41	0.000	0.538	0.684
	Constant	-0.624	0.319	-1.95	0.051	-1.249	0.002
Suicide attempts[b]	Factor loading	1.095	0.137	8.00	0.000	0.827	1.364
NSSI incidents[b]	Factor loading	0.822	0.117	7.02	0.000	0.593	1.052
No. of diagnoses[b]	Factor loading	1.674	0.177	9.48	0.000	1.328	2.021
		Loadings of predictors on the factor					
		Coeff.	*SE*	z	P	95% CI	
PPS group variable: Dummy variable 1[c]	Factor loading	0.811	0.211	3.85	0.000	0.398	1.225
PPS group variable: Dummy variable 2[c]	Factor loading	0.523	0.140	3.75	0.000	0.250	0.797
PPS group variable: Dummy variable 3[c]	Factor loading	1.198	0.172	6.97	0.000	0.861	1.535
Age	Factor loading	0.106	0.033	3.23	0.001	0.042	0.171
Sex[d]	Factor loading	-0.831	0.127	-6.52	0.000	-1.080	-0.581
Dataset[e]	Factor loading	0.252	0.103	2.45	0.014	0.051	0.453

	Error variances			
	Coeff.	*SE*	95% CI	
Factor (P)	1.000	(constrained)	-	-
Depression severity[a]	0.282	0.031	0.226	0.351
Psychosocial functioning[a]	0.574	0.041	0.498	0.661
Stress[a]	0.623	0.044	0.542	0.716
Personality functioning[a]	0.509	0.039	0.438	0.591

Notes. N = 506.

[a] Standardized effect of continuous variable. [b] Non-standardized effect of ordinal variable. Coefficient in log odds calculated using ordered logistic regression. [c] The ordinally scaled PPS group variable was dummy-coded (see coding in Table 6). [d] Sex coded as 0 = female, 1 = male. [e] Sample coded as 0 = AtR!Sk, 1 = BeBaDoc.

Table 2
Results of the best-fitting 3-factor GSEM

		Loadings of the factors (F1, F2, F3) on the indicators					
		Coeff.	*SE*	z	P	95% CI	
Depression severity[a]	Factor loading (F1)	0.421	0.047	9.02	0.000	0.329	0.512
	Constant (F1)	-0.799	0.393	-2.03	0.042	-1.570	-0.029
Psychosocial functioning[a]	Factor loading (F1)	-0.314	0.037	-8.38	0.000	-0.387	-0.240
	Constant (F1)	0.595	0.296	2.01	0.045	0.014	1.176
Stress[a]	Factor loading (F3)	1.000	(constrained)	-	-	-	-
	Constant (F3)	-0.639	0.422	-1.51	0.130	-1.466	0.188
Personality functioning[a]	Factor loading (F1)	0.346	0.039	8.91	0.000	0.270	0.422
	Constant (F1)	-0.658	0.326	-2.02	0.043	-1.296	-0.020
Suicide attempts[b]	Factor loading (F2)	1.000	(constrained)	-	-	-	-
NSSI incidents[b]	Factor loading (F2)	2.228	0.643	3.46	0.001	0.967	3.489
No. of diagnoses[b]	Factor loading (F1)	1.000	(constrained)	-	-	-	-
		Loadings of predictors on the factor F1					
		Coeff.	*SE*	z	P	95% CI	
PPS group variable: Dummy variable 1[c]		1.564	0.415	3.77	0.000	0.752	2.377
PPS group variable: Dummy variable 2[c]		0.789	0.263	3.00	0.003	0.273	1.306
PPS group variable: Dummy variable 3[c]		2.097	0.372	5.64	0.000	1.369	2.825
Age		0.172	0.061	2.83	0.005	0.053	0.292
Sex[d]		-1.331	0.256	-5.19	0.000	-1.833	-0.829
Dataset[e]		0.808	0.202	3.99	0.000	0.411	1.204

	Loadings of predictors on the factor F2					
	Coeff.	*SE*	z	P	95% CI	
PPS group variable: Dummy variable 1[c]	-0.174	0.267	-0.65	0.515	-0.698	0.350
PPS group variable: Dummy variable 2[c]	0.664	0.222	3.00	0.003	0.230	1.099
PPS group variable: Dummy variable 3[c]	0.983	0.313	3.14	0.002	0.369	1.597
Age	0.052	0.047	1.11	0.269	-0.040	0.143
Sex[d]	-1.028	0.216	-4.76	0.000	-1.451	-0.605
Dataset[e]	-0.907	0.160	-5.68	0.000	-1.219	-0.594
	Loadings of predictors on the factor F3					
	Coeff.	*SE*	z	P	95% CI	
PPS group variable: Dummy variable 1[c]	0.540	0.169	3.19	0.001	0.208	0.872
PPS group variable: Dummy variable 2[c]	0.438	0.112	3.92	0.000	0.219	0.657
PPS group variable: Dummy variable 3[c]	0.604	0.135	4.46	0.000	0.338	0.869
Age	0.095	0.027	3.57	0.000	0.043	0.147
Sex[d]	-0.691	0.100	-6.89	0.000	-0.888	-0.494
Dataset[e]	-0.307	0.082	-3.75	0.000	-0.468	-0.147

	Error variances				
	Coeff.	*SE*	z	P	95% CI	
Factor F1	3.169	0.677	-	-	2.086	4.816
Factor F2	0.841	0.325	-	-	0.394	1.795
Factor F3[e]	0.323	5.362	-	-	2.51E-15	4.17E+13
Covariance of factor error variances: F1 and F3	0.934	0.131	7.13	0.000	0.677	1.191
Covariance of factor error variances: F1 and F2	1.277	0.306	4.17	0.000	0.677	1.878
Covariance of factor error variances: F3 and F2	0.277	0.081	3.43	0.001	0.119	0.436
Depression severity[a]	0.267	0.031	-	-	0.213	0.335
Psychosocial functioning[a]	0.590	0.042	-	-	0.513	0.678
Stress[a,e]	0.480	5.362	-	-	1.52E-10	1.52E+09
Personality functioning[a]	0.502	0.038	-	-	0.432	0.583
	Correlations between factors					
	Coeff.	*SE*	z	P		
F1 and F3	0.585	0.035	16.50	0.000		
F1 and F2	0.780	0.098	7.98	0.000		
F3 and F2	0.336	0.074	4.55	0.000		

Notes. *N* = 506. F1 = Psychopathology and functional impairment, F2 = Self-harming behavior, F3 = Perceived Stress.
[a] Standardized effect of continuous variable. [b] Non-standardized effect of ordinal variable. Coefficient in log odds calculated using ordered logistic regression. [c] The ordinally scaled PPS group variable was dummy-coded (see coding in Table 6). [d] Sex coded as 0 = female, 1 = male. [e] Sample coded as 0 = AtR!Sk, 1 = BeBaDoc. [e] Error variance is not interpretable because the GSEM module of Stata does not allow the necessary parameter restriction of the third factor. To obtain interpretable error variances and test the validity of the final model structure, an equivalent model was fitted in Mplus (see next table).

Table 3
Results of the model computed in Mplus corresponding to the best-fitting 3-factor GSEM, with Stress included as manifest variable in the factor structure

		Loadings of the factors (F1, F2) on the indicators			
		Coeff.	*SE*	z	Two-Tailed P-Value (95%)
Depression severity[a]	Factor loading (F1)	0.422	0.047	9.03	0.000
Psychosocial functioning[a]	Factor loading (F1)	-0.315	0.038	-8.38	0.000
Personality functioning[a]	Factor loading (F1)	0.347	0.039	8.91	0.000
Suicide attempts[b]	Factor loading (F2)	1.000	(constrained)	-	-
NSSI incidents[b]	Factor loading (F2)	2.227	0.646	3.45	0.001
No. of diagnoses[b]	Factor loading (F1)	1.000	(constrained)	-	-
		Intercepts			
Depression severity[a]		-0.242	0.063	-3.82	0.000
Psychosocial functioning[a]		0.180	0.057	3.18	0.001
Personality functioning[a]		-0.200	0.059	-3.40	0.001
Stress[a]		0.138	0.066	2.09	0.037
		Loadings of predictors on the factor F1			
		Coeff.	*SE*	z	Two-Tailed P-Value (95%)
PPS group variable: Dummy variable 1[c]		1.558	0.413	3.78	0.000
PPS group variable: Dummy variable 2[c]		0.788	0.262	3.00	0.003
PPS group variable: Dummy variable 3[c]		2.089	0.370	5.65	0.000
Age		0.172	0.061	2.83	0.005
Sex[d]		-1.324	0.255	-5.19	0.000
Dataset[e]		0.806	0.201	4.00	0.000

	Loadings of predictors on the factor F2			
	Coeff.	*SE*	z	Two-Tailed P-Value (95%)
PPS group variable: Dummy variable 1[c]	-0.174	0.269	-0.65	0.516
PPS group variable: Dummy variable 2[c]	0.664	0.222	2.99	0.003
PPS group variable: Dummy variable 3[c]	0.985	0.315	3.13	0.002
Age	0.052	0.047	1.11	0.268
Sex[d]	-1.032	0.217	-4.75	0.000
Dataset[e]	-0.911	0.161	-5.67	0.000
	Loadings of predictors on Stress (manifest variable)			
	Coeff.	*SE*	z	Two-Tailed P-Value (95%)
PPS group variable: Dummy variable 1[c]	0.540	0.169	3.19	0.001
PPS group variable: Dummy variable 2[c]	0.438	0.112	3.92	0.000
PPS group variable: Dummy variable 3[c]	0.604	0.135	4.46	0.000
Age	0.095	0.027	3.58	0.000
Sex[d]	-0.691	0.100	-6.88	0.000
Dataset[e]	-0.307	0.082	-3.74	0.000

	Error variances			
	Coeff.	*SE*	z	Two-Tailed P-Value (95%)
Factor F1	3.141	0.670	4.69	0.000
Factor F2	0.855	0.332	2.58	0.010
Stress[a]	0.804	0.051	15.75	0.000
Covariance of factor error variances: F1 and Stress	0.930	0.130	7.13	0.000
Covariance of factor error variances: F1 and F2	1.277	0.307	4.16	0.000
Covariance of factor error variances: Stress and F2	0.279	0.082	3.42	0.001
Depression severity[a]	0.267	0.031	8.61	0.000
Psychosocial functioning[a]	0.590	0.042	14.06	0.000
Personality functioning[a]	0.502	0.038	13.04	0.000
	Correlations			
	Coeff.	*SE*	z	Two-Tailed P-Value (95%)
F1 and Stress	0.585	0.035	16.50	0.000
F1 and F2	0.780	0.098	7.98	0.000
Stress and F2	0.336	0.074	4.55	0.000

Notes. $N = 506$. F1 = Psychopathology and functional impairment, F2 = Self-harming behavior. The variable *Stress* was included into the factor structure as manifest variable.

[a] Standardized effect of continuous variable. [b] Non-standardized effect of ordinal variable. Coefficient in log odds calculated using ordered logistic regression. [c] The ordinally scaled PPS group variable was dummy-coded (see coding in Table 6). [d] Sex coded as 0 = female, 1 = male. [e] Sample coded as 0 = AtR!Sk, 1 = BeBaDoc.

Table 4

Results of Ordered Logistic Regression with NSSI as outcome (additional exploration of hypothesis 3)

	Odds ratio	*SE*	z	P	95% CI	
PPS group variable: Dummy coded variable 1[a]	0.690	0.270	-0.95	0.342	0.320	1.484
PPS group variable: Dummy coded variable 2[a]	2.960	1.069	3.01	0.003	1.459	6.007
PPS group variable: Dummy coded variable 3[a]	3.205	1.362	2.74	0.006	1.393	7.373
Sex[b]	0.191	0.047	-6.71	0.000	0.118	0.310
Age	1.047	0.078	0.61	0.542	0.904	1.212
Dataset[c]	0.144	0.035	-7.86	0.000	0.089	0.233
Log Likelihood = -323.523						
Chi^2 (6) = 148.03, $p < 0.001$						
Pseudo $R^2 = 0.186$						
	Contrast (group comparison)					
	Odds ratio	*SE*	Chi^2	P	95% CI	
delusions only vs. hallucinations only	4.292	2.150	2.910	0.004	1.609	11.455

Notes. N = 499.

[a]The ordinally scaled PPS group variable was dummy-coded (see coding in Table 6). [b] Sex coded as 0 = female, 1 = male. [c] Sample coded as 0 = AtR!Sk, 1 = BeBaDoc.

Table 5

Results of Ordered Logistic Regression with suicide attempts as outcome (additional exploration of hypothesis 3)

	Odds ratio	*SE*	z	P	95% CI	
PPS group variable: Dummy coded variable 1[a]	1.050	0.453	0.11	0.909	0.451	2.448
PPS group variable: Dummy coded variable 2[a]	2.228	0.555	3.21	0.001	1.367	3.632
PPS group variable: Dummy coded variable 3[a]	3.568	1.072	4.23	0.000	1.980	6.431
Sex[b]	0.606	0.156	-1.95	0.051	0.366	1.003
Age	1.145	0.075	2.06	0.039	1.007	1.302
Dataset[c]	1.107	0.219	0.52	0.605	0.752	1.631
Log Likelihood = -400.501						
Chi^2 (6) = 31.86, $p < 0.001$						
Pseudo R^2 = 0.0383						
	Contrast (group comparison)					
	Odds ratio	*SE*	Chi^2	P	95% CI	
delusions only vs. hallucinations only	2.121	0.994	2.580	0.108	0.847	5.313

Notes. N = 500.

[a]The ordinally scaled PPS group variable was dummy-coded (see coding in Table 6). [b] Sex coded as 0 = female, 1 = male. [c] Sample coded as 0 = AtR!Sk, 1 = BeBaDoc.

Table 6
Dummy coding of the PPS group variable

	Dummy variable 1	Dummy variable 2	Dummy variable 3	Cases
No occurrence of positive psychotic symptoms (noPPS)	0	0	0	341
Only delusional beliefs (del)	1	0	0	32
Only hallucinations (hall)	0	1	0	80
Delusional beliefs and hallucinations (del&hall)	0	0	1	53

Notes. $N = 506$.

Group comparisons of the factor values of the best-fitting multifactorial model: An illustration of the magnitude of the contrasts

Hypothesis 1: noPPS vs. PPS

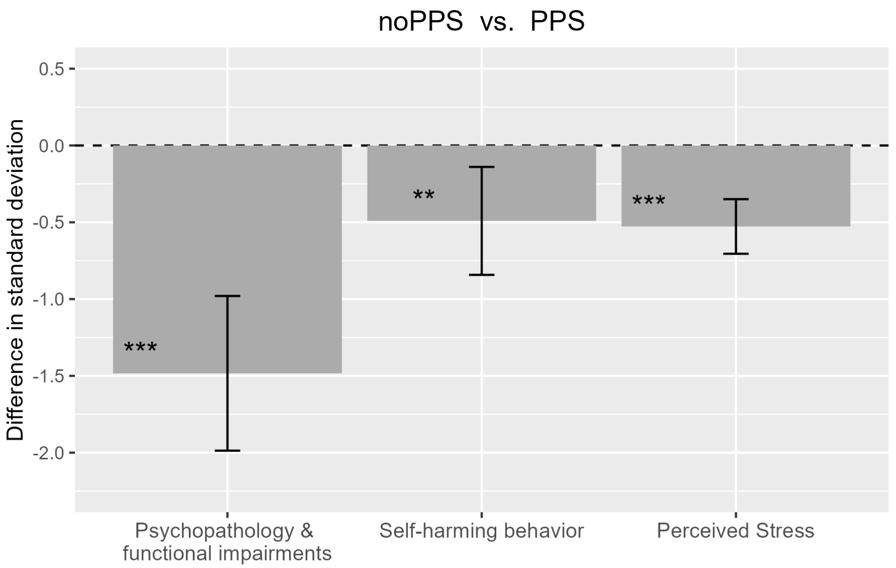

Notes. This figure illustrates the difference in standard deviation from the estimated factor values for the linear combination of the dummy-coded PPS group variable. (no)PPS = (no) occurrence of positive psychotic symptoms. The whiskers represent the confidence interval of the contrast. *** indicates p < .001, ** indicates p <.01, * indicates p <.05.

Hypothesis 1: noPPS vs. del

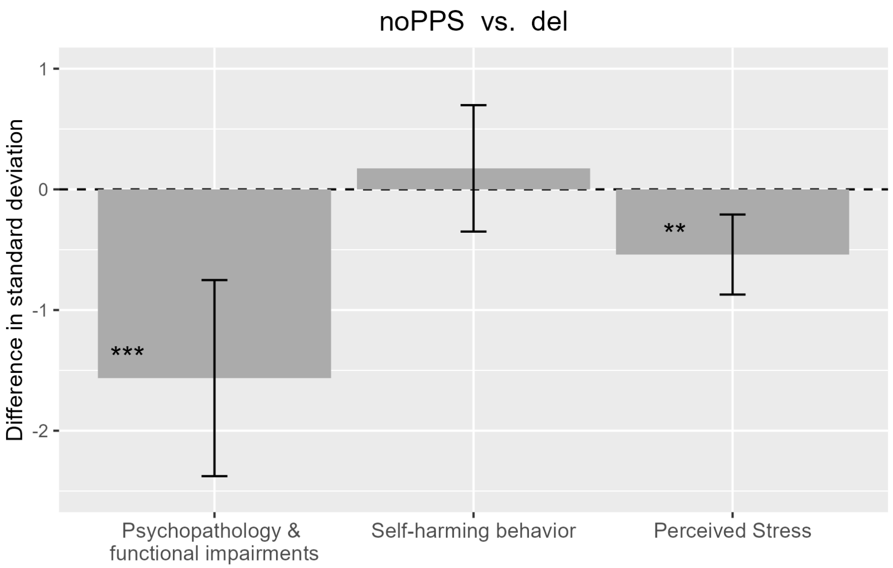

Notes. This figure illustrates the difference in standard deviation from the estimated factor values for the linear combination of the dummy-coded PPS group variable. noPPS = no occurrence of positive psychotic symptoms, del = delusional beliefs only. The whiskers represent the confidence interval of the contrast. *** indicates p < .001, ** indicates p <.01, * indicates p <.05.

Hypothesis 1: noPPS vs. hall

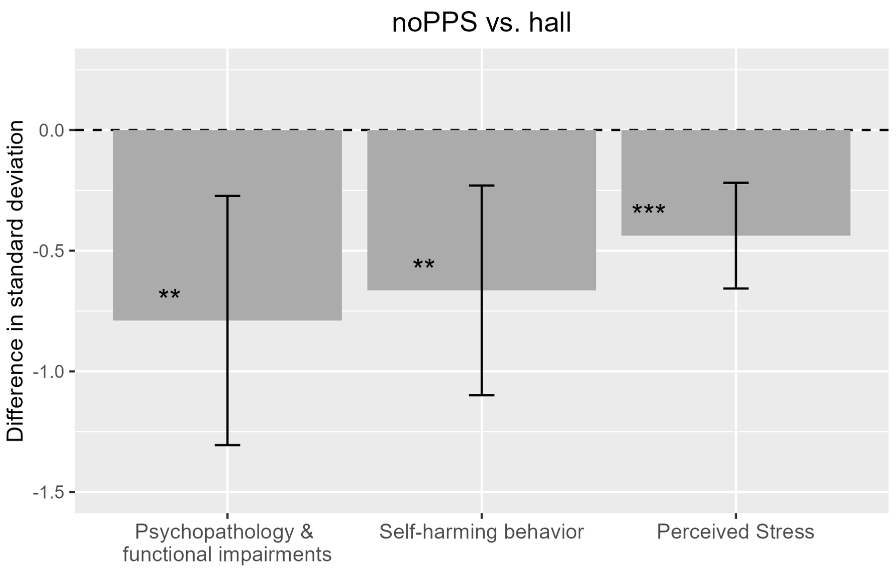

Notes. This figure illustrates the difference in standard deviation from the estimated factor values for the linear combination of the dummy-coded PPS group variable. noPPS = no occurrence of positive psychotic symptoms, hall = hallucinations only. The whiskers represent the confidence interval of the contrasts. *** indicates p < .001, ** indicates p <.01, * indicates p <.05.

Hypothesis 2: del vs. del&hall

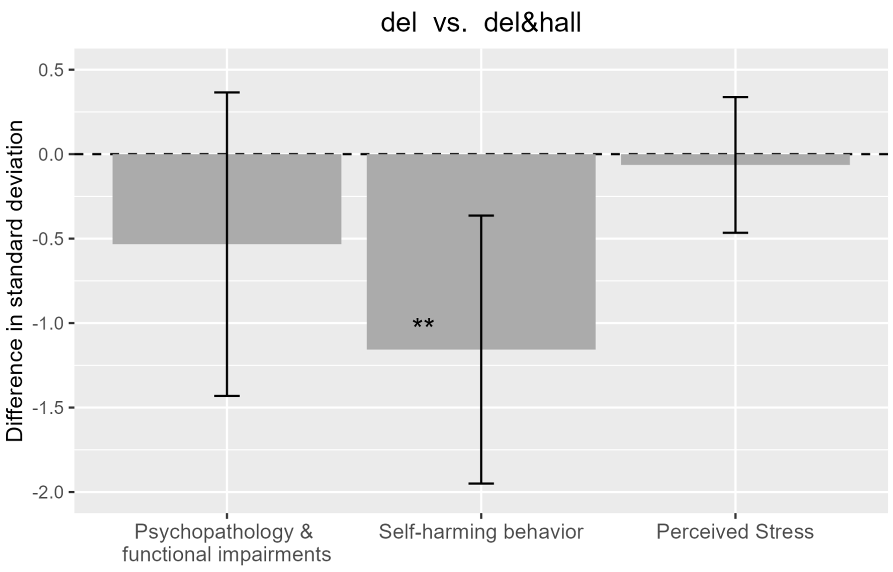

Notes. This figure illustrates the difference in standard deviation from the estimated factor values for the linear combination of the dummy-coded PPS group variable. del = delusional beliefs only, del&hall = delusional beliefs and hallucinations. The whiskers represent the confidence interval of the contrasts. *** indicates p < .001, ** indicates p <.01, * indicates p <.05.

Hypothesis 2: hall vs. del&hall

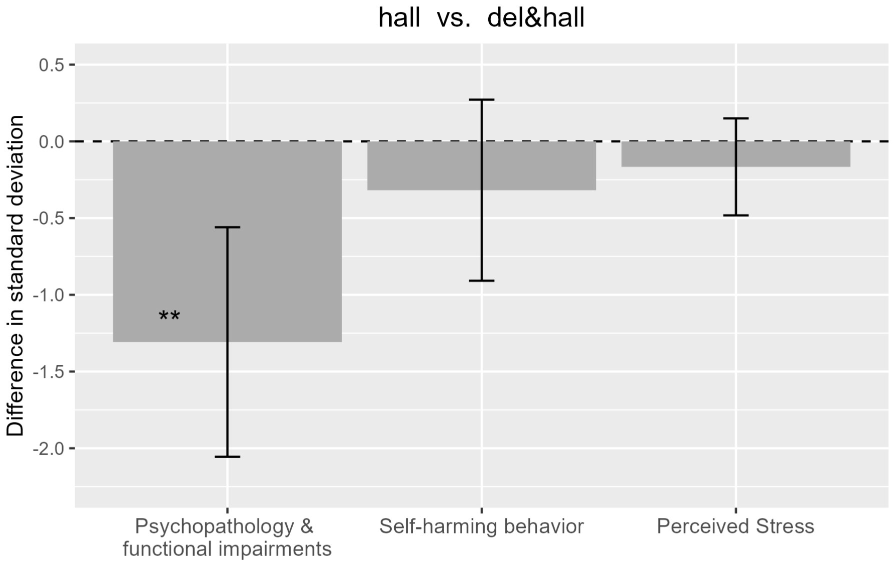

Notes. This figure illustrates the difference in standard deviation from the estimated factor values for the linear combination of the dummy-coded PPS group variable. hall = hallucinations only, del&hall = delusional beliefs and hallucinations. The whiskers represent the confidence interval of the contrasts. *** indicates p < .001, ** indicates p <.01, * indicates p <.05.

Hypothesis 3: del vs. hall

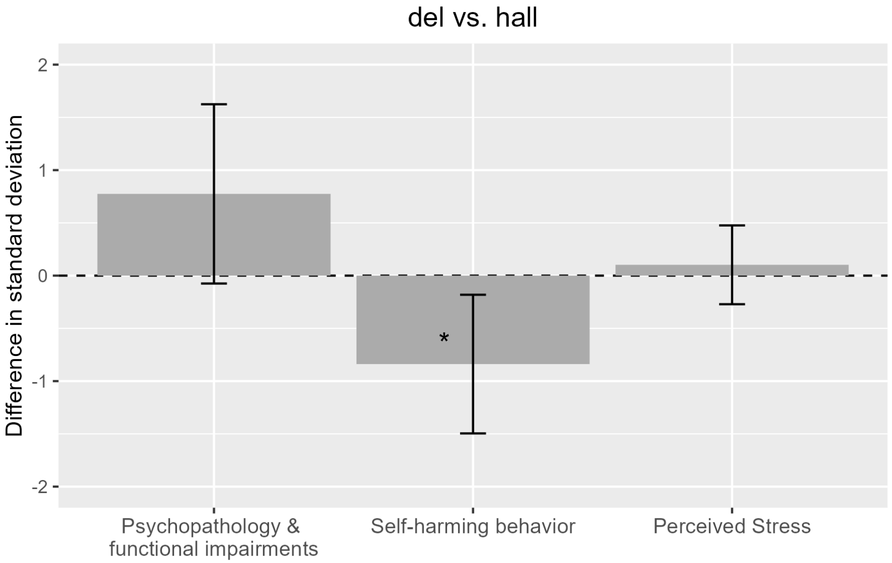

Notes. This figure illustrates the difference in standard deviation from the estimated factor values for the linear combination of the dummy-coded PPS group variable. del = delusional beliefs only, hall = hallucinations only. The whiskers represent the confidence interval of the contrasts. *** indicates p < .001, ** indicates p <.01, * indicates p <.05.

Items of the Mini International Neuropsychiatric Interview for Children and Adolescents (MINI-KID; Sheehan et al., 2010) used to assess positive psychotic symptoms (translated from German to English)

- R1: Persecutory delusions, “Have you ever believed that people are secretly watching you? Have you ever thought that someone was trying to get you? Or to hurt you?”
- R2: Mind reading, “Have you ever believed that someone can read your mind? Or that someone can hear your thoughts? Or that you can actually read someone else's thoughts? Or that you can hear what others are thinking?”
- R3: Thought insertion and delusions of control, “Have you ever thought that someone or something has put thoughts into your mind that were not your own? Did you think that someone or something has made you act in a way that is not normally not your typical self? Have you ever thought that you are obsessed about something?”
- R4: Referential ideas, “Have you ever believed that someone is sending you special messages via radio, internet, television or the news? Or through books, games or your toys? Have you ever thought that someone you didn't know was particularly interested in you?”
- R5: Strange beliefs, “Have your family or friends ever thought that any of your beliefs were strange or peculiar? Please give an example.”
- R6: Auditory hallucinations, “Have you ever heard things that others could not hear, such as voices? Have you heard a voice talking about your thoughts or behavior? Have you heard several voices talking to each other?”
- R7: Visual hallucinations, “Have you ever had visions or seen things that others could not see?”

Reference:

Sheehan DV, Sheehan KH, Shytle RD, et al (2010) Reliability and Validity of the Mini International Neuropsychiatric Interview for Children and Adolescents (MINI-KID). J Clin Psychiatry 71:313–326. https://doi.org/10.4088/JCP.09m05305whi
